# Survie et facteurs pronostiques du cancer bronchique non à petites cellules chez le sujet jeune au centre tunisien

**DOI:** 10.11604/pamj.2020.35.19.21100

**Published:** 2020-01-23

**Authors:** Samah Joobeur, Ahmed Ben Saad, Asma Migaou, Nesrine Fahem, Saousen Cheikh Mhamed, Naceur Rouatbi

**Affiliations:** 1 Service de Pneumologie et d’Allergologie, Hôpital Universitaire Fattouma Bourguiba, Rue du 1^er^ Juin 1955, Monastir 5000, Tunisie

**Keywords:** Cancer bronchique primitif, cancer bronchique non à petites cellules, pronostic, survie, Primary lung cancer, small cell lung cancer, prognosis, survival

## Abstract

**Introduction:**

Le carcinome bronchique non à petites cellules (CBNPC) constitue un problème de santé publique qui touche classiquement le sujet âgé. Actuellement et depuis quelques années, cette pathologie s’étend de plus en plus vers la population jeune. L’objectif de ce travail est d’étudier les caractéristiques du CBNPC chez le sujet jeune et d’évaluer sa survie ainsi que les différents facteurs pronostiques.

**Méthodes:**

Il s’agit d’une étude rétrospective ayant inclus tous les patients âgés de moins de 50 ans, pris en charge au service de pneumologie du Centre Hospitalo-Universitaire (CHU) Fattouma Bourguiba de Monastir pour CBNPC. La survie et les facteurs pronostiques ont été analysés selon la méthode de Kaplan Meier.

**Résultats:**

L’âge moyen de nos patients était de 43,8 ± 5,29 ans. Le type histologique le plus fréquent était l’adénocarcinome bronchique (66,1%). Le CBNPC a été découvert à un stade localement avancé ou métastatique dans 79,7% des cas. La médiane de survie était de 8 ± 0,72 mois. En analyse univariée, l’état général des patients évalué par l’indice “Performance Status” (PS) de l’OMS à l’admission, le stade de la tumeur et la protéine C-réactive (CRP) ont influencé de façon significative la survie. L’analyse multivariée a permis de retenir un indice PS ≥ 2 et une CRP élevée comme facteurs de mauvais pronostic.

**Conclusion:**

Malgré les progrès thérapeutiques, le CBNPC du sujet jeune demeure de mauvais pronostic. Un diagnostic et une prise en charge précoces permettent d’améliorer la survie de ces patients.

## Abstract (en)

**Introduction:**

Non-small cell lung cancer (NSCLC) is a public health problem that usually affects the elderly. Currently and for some years now, this disease is increasingly affecting the young population. The purpose of this study was to analyze the features of NSCLC in young subjects and to assess survival as well as the various prognostic factors.

**Methods:**

We conducted a retrospective study of all patients under the age of 50 years treated in the Department of Pneumology at the Fattouma Bourguiba University Hospital, Monastir for NSCLC. Survival and prognostic factors have been analyzed according to Kaplan Meier method.

**Results:**

The average age of patients was 43.8 ± 5.29 years. The most common histological type was lung adenocarcinoma (66.1%). NSCLC was discovered at an advanced or metastatic stage in 79.7% of cases. The median overall survival was 8 ± 0.72 months. Univariate analysis showed that survival was significantly influenced by patients’ general status, assessed according to the “Performance Status (PS)” index of the World Health Organization on admission, tumor stage and CRP concentrations. Multivariate analysis was performed, which enabled us to use PS index ≥ 2 and high CRP concentrations as factors of poor prognosis.

**Conclusion:**

Despite the therapeutic progress, prognosis in young subjects with NSCLC is poor. Early diagnosis and management can improve survival in these patients.

## Introduction

Le cancer broncho-pulmonaire primitif (CBP) constitue un problème de santé publique de part sa fréquence croissante et son pronostic réservé. Il est le premier cancer chez l’homme et le quatrième chez la femme. Son incidence mondiale en 2012 était estimée à 1,8 millions de cas soit 12,9% de tous les cancers. Il représente la première cause de mortalité par cancer chez l’homme et la deuxième cause chez la femme, responsable de 1,59 millions de décès [1]. En Tunisie, il est le premier cancer chez l’homme [2]. Son incidence standardisée est passé de 26,3 cas/100000 habitants en 2003 à 33,5 cas/100000 habitants en 2008 et sera de 48,5 cas/100000 selon les projections 2019-2024 [2]. Les cancers bronchiques non à petites cellules (CBNPC) représentent plus de 80% des CBP [3]. Malgré les progrès diagnostiques et thérapeutiques, le pronostic reste réservé avec une survie à 5 ans inférieure à 15% tous stades confondus [3]. Le CBP survient plus fréquemment entre la 6^ème^ et la 8^ème^ décennie et il est relativement rare chez les patients plus jeunes [4,5]. Seuls 5% à 12,5% des cancers du poumon sont diagnostiqués chez des patients âgés de moins de 50 ans [6]. Plusieurs facteurs plus ou moins associés semblent expliquer la survenue du CBP à un âge jeune, en particulier un âge de début précoce de l’intoxication tabagique, mais aussi des facteurs environnementaux, professionnels et génétiques [3,7-12]. A côté de ce profil étiopathogénique particulier au CBP chez les jeunes, ce cancer présente également des caractéristiques démographiques, cliniques et évolutives différentes comparativement au CBP dans la population générale [7,9,13,14]. L’objectif de ce travail est d’étudier les caractéristiques démographiques, cliniques et thérapeutiques du CBNPC chez le sujet jeune et d’évaluer sa survie ainsi que les différents facteurs pronostiques.

## Méthodes

**Type de l’étude:** il s’agit d’une étude rétrospective portant sur les dossiers des patients âgés de moins de 50 ans porteurs de CBNPC pris en charge au service de pneumo-allergologie du CHU Fattouma Bourguiba de Monastir entre janvier 1990 et décembre 2016.

**Population d’étude:** les critères d’inclusion etaient: les patients chez lesquels le diagnostic du CBNPC a été confirmé histologiquement; age inférieure à 50 ans et tous les stades ont été inclus. Les critères d’exclusion etaient: tous les cas dont le diagnostic n’a pas été confirmé histologiquement; carcinome bronchique à petites cellules et cancer pulmonaire secondaire.

**Recueil de données:** nous avons noté pour chaque malade: l’âge, le sexe, le tabagisme, les antécédents respiratoires et extra-respiratoires et les signes fonctionnels. Le bilan initial a comporté: un examen physique complet; un bilan biologique initial comportant: une numération de la formule sanguine (NFS), un ionogramme sanguin, la protéine C-réactive (CRP) et des marqueurs tumoraux; une radiographie du thorax; une fibroscopie bronchique en dehors des contres indications; un bilan d’extension comportant un scanner thoraco-abdominal ± cérébral; d’autres examens sont demandés en fonction de l’orientation clinique et des résultats du bilan initial; l’établissement d’une stadification de la maladie. Nous avons aussi précisé les modalités thérapeutiques ainsi que l’évolution de la maladie et la survie.

**Classification et évaluation:** l’état général des patients était évalué par l’indice de performance status (PS) de l’OMS (Tableau 1). Au terme du bilan initial une classification « Tumor Node Metastasis (TNM) » selon la 7^ème^ édition a été établie pour chaque malade [15].

**Tableau 1:** évaluation de l’indice de “Performance Status” de l’OMS

PS	Description
0	Capable d’une activité identique à celle précédent la maladie sans aucune restriction
1	Activité physique diminuée mais ambulatoire et capable de mener un travail
2	Ambulatoire et capable de prendre soins de soi-même; incapable de travailler; alité moins de 50% de son temps
3	Capable seulement de quelques soins; alité ou en chaise plus de 50% du temps
4	Incapable de prendre soins de soi-même; alité ou en chaise en permanence

**Survie:** le recueil des données concernant la survie des patients s’est basé sur les données des dossiers médicaux pour les patients décédés à l’hôpital et sur le contact téléphonique avec la famille pour les patients décédés à domicile.

**Analyse statistique et étude des facteurs pronostiques:** les données ont été saisies et analysées par le logiciel SPSS version 20.0. Les variables quantitatives ont été exprimées en moyennes ± déviations standard. Les variables qualitatives ont été exprimées en pourcentages. L’analyse de la survie a été réalisée par la méthode de Kaplan-Meier. La comparaison de la survie en fonction des différentes variables pronostiques a été faite par le test du Log-Rank en analyse univariée. L’analyse multivariée a été effectuée par le modèle de Cox pour identifier les facteurs indépendants. Le modèle a inclus toutes les variables avec une valeur de p < 0,2 en analyse univariée. Pour tous les tests statistiques, le seuil de signification statistique a été fixé à 0,05.

## Résultats

**Caractéristiques de la population d’étude:** entre janvier 1990 et décembre 2016, 751 patients porteurs du CBNPC ont été hospitalisés et pris en charge dans notre service. Parmi ces patients, 118 (15,7%) avaient un âge inférieur à 50 ans. L’âge de nos patients variait entre 23 ans et 49 ans avec une moyenne de 43,83 ± 5,29 ans. Vingt-six patients (22%) avaient un âge ≤ 40 ans. Il existait une nette prédominance masculine avec 108 hommes (91,5%) contre 10 femmes (8,5%) soit un sexe ratio de 10,8. Cent-cinq patients (89%) étaient des fumeurs avec une consommation tabagique moyenne de 32,9 paquets-années (PA). Les antécédents personnels étaient dominés par la broncho-pneumopathie chronique obstructive (BPCO) retrouvée chez 12 patients (10,2% des cas). Des antécédents familiaux de cancer ont été notés chez 10 patients. Le délai moyen écoulé entre l’apparition des premiers signes fonctionnels et la première consultation était de 74 jours. La douleur thoracique était le symptôme le plus fréquent, retrouvée chez 70 patients (59,3%). L’altération de l’état général était la principale manifestation extra-respiratoire notée dans 67 observations (56,8%). Dans 4 cas, la découverte du CBNPC était fortuite. L’indice PS de l’OMS était inférieur à 2 chez 99 patients (83,9%). Tous les patients avaient une confirmation histologique. Elle a été obtenue essentiellement par fibroscopie bronchique dans 44,9% des cas et par ponction biopsie trans-pariétale dans 26,2% des cas. Chez 14 patients on a eu recours à la chirurgie pour confirmer le diagnostic. Il s’agissait d’un adénocarcinome dans 78 cas (66,1%), un carcinome épidermoïde dans 34 cas (28,8%), et un carcinome à grande cellules dans 4 cas (3,4%). D’autres types histologiques plus rares ont été notés dans le reste de la population. Le Tableau 2 résume les caractéristiques de notre population.

**Tableau 2:** Principales caractéristiques de la population d’étude

	Nombre, Moyenne (±DS)	%
**Age (ans)**	43,83 ± 5,29	
**Genre (M)**	108	91,5
**Tabagisme:**		
- Absent	13	11
- Sevré	17	14,4
- Actif	88	74,6
**Intoxication tabagique (PA)**	32,9 ± 21,5	
**Durée d’évolution des symptômes (j)**	74,2	
**Signes fonctionnels:**		
- Douleur thoracique	70	59,3
- Toux	51	43,2
- Hémoptysie	36	30,5
- Altération de l’état général	67	56,8
**Types histologiques:**		
- Adénocarcinome	78	66,1
- Carcinome épidermoide	34	28,8
- Carcinome à grandes cellules	4	3,4
- Autres	2	1,7
**Stades du CBNPC: (TNM)**		
- I	5	4,2
- II	5	4,2
- IIIA	14	11,9
- IIIB	14	11,9
- IV	80	67,8

M: masculin, PA: paquets-années, j: jours, TNM: classification TNM (T: tumeur primitive, N: node ou ganglion, M: métastases)

**Stadification et traitement:** le bilan d’extension et la classification TNM ont conclu à un stade I, II et IIIA chez 4,2%, 4,2% et 11,9% des patients respectivement. Un stade localement avancé (IIIB) a été noté dans 11,9% des cas et un stade IV dans 67,8% des cas. Sur le plan thérapeutique, 95 patients (80,5%) ont bénéficié d’un traitement anticancéreux spécifique basé sur la chirurgie (24 patients soit 20,3%) et/ou la radiothérapie (27 patients soit 23%) et/ou la chimiothérapie (88 patients soit 74,6%).

### Survie et facteurs pronostiques

**Figure 1:**
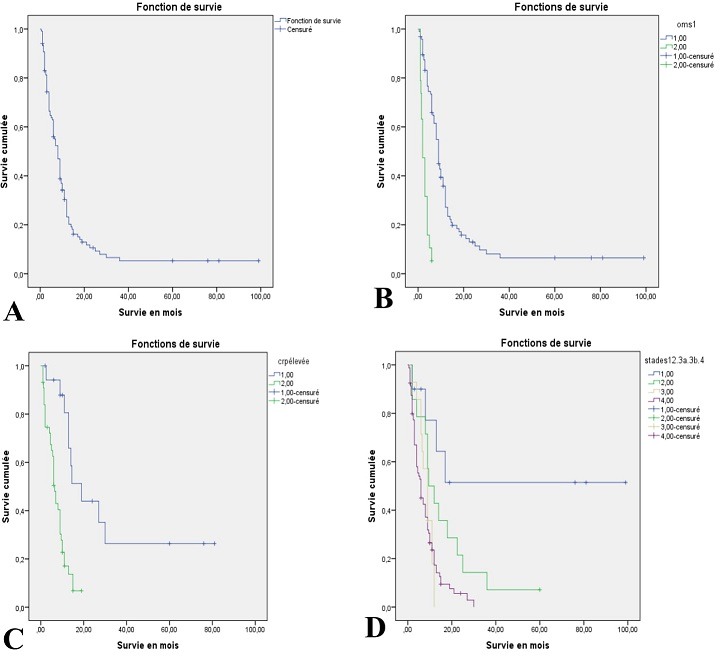
Courbes de survie; A: courbe de survie globale, B: survie en fonction du score PS de l’OMS; 1: score PS de OMS ≤1; 2: score PS de OMS ≥2, C: survie en fonction de la CRP; 1: CRP normale; 2: CRP élevée, D: survie selon les stades TNM; 1: stade I et II; 2: stade IIIA; 3: stade IIIB; 4: stade IV

**Survie globale:** jusqu’à décembre 2016, 102 patients étaient décédés, 11 étaient encore en vie et 5 étaient perdus de vue. La médiane de survie de nos patients était 8 ± 0,72 mois [6,59 - 9,41]. La survie à 1 an, 2 ans et à 3 ans était respectivement de 23,2%, 9,2% et 5,3% (Figure 1).

### Facteurs influençant la survie

**Etude univariée:** nous avons tenu en considération différents paramètres cliniques et biologiques, le type histologique ainsi que le stade de la tumeur dans l’analyse univariée. L’état général des patients évalué par l’indice PS de l’OMS à l’admission, le stade de la tumeur et la CRP ont influencé de façon significative la survie (Tableau 3). En effet, la médiane de survie des patients ayant un indice PS de l’OMS-1 était de 9 mois contre 2 mois pour les patients ayants un score ≥ 2. De même, un taux élevé de la CRP (≥ 7 mg/l) a constitué un facteur du mauvais pronostic chez nos patients avec p<0,001. Les patients ayant une tumeur localisée avaient une meilleure survie que ceux ayant une tumeur localement avancée ou métastatique avec une différence statistiquement significative (Figure 1). L’âge, le sexe, le tabagisme, le type histologique, les données de la NFS, n’ont pas constitué de facteurs pronostiques pour notre population (Tableau 3).

**Tableau 3:** Facteurs pronostiques du cancer bronchique non à petites cellules chez le sujet jeune: analyse univariée

Facteurs pronostiques	Nombre	Médiane de survie (mois)	p
**Facteurs cliniques:**			
Age:			
≤ 40 ans	26	4 ± 2,06	0,822
> 40 ans	92	8 ± 0,82	
Genre:			
Hommes	108	8 ± 0,69	0,294
Femmes	10	6 ± 4,9	
Tabagisme:			
Tabagiques	105	8 ± 0,68	0,7
Non tabagiques	13	4 ± 2,39	
Fumeurs:			
Non sevrés	88	8 ± 0,9	0,257
Sevrés	17	9 ± 0,9	
Indice PS:			
PS ≤ 1	96	9 ± 0,63	**<0,001**
PS > 2	19	2 ± 0,54	
**Facteurs biologiques:**			
CRP:			
Normale	18	19 ± 4,5	**<0,001**
Elevée	44	6,5 ± 0,6	
Anémie:			
Oui	42	6 ± 1,05	0,236
Non	70	9 ± 0,66	
Thrombocytose:			
Oui	60	6 ± 1,05	0,107
Non	47	9 ± 0,95	
**Types Histologiques:**			
Carcinome épidermoide	34	7 ± 2,4	0,596
Adénocarcinome	78	8 ± 0,8	
**Stades TNM:**			
I	5	19 ± 1,62	**<0,001**
II	5	9 ± 1,77	
IIIA	14	10 ± 0,98	
IIIB	14	7 ± 0,66	
IV	80	5 ± 0,33	

**Etude multivariée:** l’analyse multivariée a permis de retenir comme facteurs de mauvais pronostique: l’altération de l’état général avec un indice PS ≥ 2 (hazard ratio (HR) = 6,195) et une CRP élevée (HR = 4,152) (Tableau 4).

**Tableau 4:** Facteurs pronostiques du cancer bronchique non à petites cellules chez le sujet jeune: analyse multivariée

Facteurs	HR	IC	p
Indice PS	6,195	2,470-15,537	0,001
CRP	4,152	1,830-9,422	0,001

## Discussion

Notre travail visait à étudier le CBNPC chez le sujet jeune en terme de caractéristiques, survie et facteurs pronostiques. Un indice PS ≥ 2 et une CRP élevée étaient les deux facteurs indépendants associés à un mauvais pronostic du CBNPC chez le sujet jeune. L’incidence du CBP ne cesse d’augmenter chaque année dans les divers pays du monde avec un taux d’accroissement annuel estimé à 3% selon l’OMS, taux le plus élevé dans toutes les affections néoplasiques. Ce cancer s’étend aux différentes tranches d’âge et affecte de plus en plus la population jeune avec des aspects cliniques et évolutifs souvent différents de ceux habituellement observés dans la population générale [7,9,13,14]. Dans notre étude ainsi que dans plusieurs autres série, 50 ans a été choisi pour définir les sujets «jeunes» ayant un CBP. Ceci parce que l’incidence de ce cancer augmente rapidement au-delà de cet âge et pour permettre une analyse statistique significative [5,6,16].

### Particularités du CBNPC chez le sujet jeune

Dans la littérature, le CBP touche dans 0,6 à 12,5% des cas les sujets jeunes [9,17-20]. Cette fréquence a tendance à augmenter avec l’expansion tabagique et l’accroissement de l’incidence de ce cancer. Dans notre série les jeunes ayant un CBNPC représentent 15,7%. Ceci pourrait être expliqué par l’importance du tabagisme chez nos jeunes avec un âge de début précoce de l’intoxication tabagique. La moyenne d’âge dans les études ayant pris une limite d’âge similaire à la notre (inférieur à 50 ans) était de 43 à 45 ans [16,19,20]. D’autre part, la majorité des cas de CBP chez les jeunes surviennent entre 40 et 50 ans. Classiquement, le CBP était considéré comme une affection qui touche de prédilection l’homme fumeur mais avec l’expansion du tabagisme dans la population féminine au cours des dernières décennies, le sexe ratio tend à s’inverser dans certaines études [21,22]. Le tabagisme est incontestablement le principal facteur du risque du CBP chez les jeunes. En effet, dans la littérature le taux de tabagisme dépasse 79% dans la majorité des études [13,17,20,22,23]. Cependant, l’intoxication tabagique n’est pas le seul facteur qui favorise l’éclosion du CBP chez le jeune. En effet, quelques études ont rapporté une faible intoxication tabagique chez les jeunes patients porteurs du CBP, et évoquant ainsi l’existence d’autres facteurs environnementaux ou professionnels qui agissent très probablement en synergie avec le tabagisme et qui rendent les jeunes plus susceptibles à l’effet carcinogène du tabac [3,7-9,24]. Des facteurs génétiques semble aussi être impliqués dans l’apparition du CBNPC chez les sujets jeunes [10-12,25].

Sur le plan histologique, l’adénocarcinome était la forme histologique la plus fréquente dans notre étude (66,1% des cas) suivi par le carcinome épidermoïde (28,8%). Dans la littérature la répartition des formes histologiques du CBP chez les sujets jeunes varie selon les critères d’inclusion mais avec toujours une prédominance de l’adénocarcinome bronchique [13,14,17,19,26]. Plusieurs arguments ont été avancés pour expliquer l’accroissement des adénocarcinomes chez les jeunes particulièrement. En effet, les modifications des habitudes tabagiques avec l’apparition des filtres et des cigarettes légères favorisent le dépôt des petites particules dans les alvéoles responsables alors des adénocarcinomes. D’autre part, le développement de méthodes diagnostiques histologiques plus performantes permettent parfois de classer des carcinomes indifférenciés en adénocarcinomes. En plus, nous notons l’amélioration des techniques diagnostiques (fibroscopes souples, biopsies trans-bronchiques, biopsie sous scanner) pour les petites tumeurs périphériques plus difficiles d’accès qui sont le plus souvent des adénocarcinomes. Du point de vue de la stadification, la majorité des auteurs rapportent une fréquence élevée des formes évoluées du CBNPC chez les jeunes au moment du diagnostic. Le pourcentage des stades IIIB et IV varie de 77 à 95,2% [14,19,20,24,26,27]. Ceci a été expliqué en partie par un retard diagnostique résultant d’un faible degré de suspicion de cancer chez ces patients [20,27-29]. Dans notre série, la majorité de nos patients (79,7%) étaient d’emblée à un stade localement invasif ou métastatique au moment du diagnostic et seulement 8,4% avaient un cancer aux stades précoces (stade I et II).

### Survie et facteurs pronostiques

Malgré les progrès thérapeutiques, le CBNPC du sujet jeune reste de mauvais pronostic. La médiane de survie varie de 5,3 à 13 mois selon les auteurs avec une survie à 5 ans inférieure à 18% [13,20,22,23,26]. Cette variabilité pourrait être expliquée par certaines différences dans les critères d’inclusion et les modalités thérapeutiques. Dans notre série, la médiane de survie était de 8 mois. Cette faible survie est essentiellement en rapport avec la découverte tardive du cancer. En effet, la majorité des patients sont déjà au stade localement invasif ou métastatique au moment du diagnostic. L’appréciation de l’état général du patient est une étape essentielle dans la prise en charge du CBNPC et conditionne la décision thérapeutique. En effet, le recours à des moyens thérapeutiques moins agressifs est recommandé devant un PS supérieur ou égal à 2 [25]. Le PS est un facteur pronostique reproductible. La survie est inversement proportionnelle au PS initial dans la majorité des études [22,30,31]. Dans l’étude de Skarin *et al.* [22], on a montré que la survie se dégrade significativement avec la sévérité du PS. Ainsi, pour les malades ayant un PS 0 ou 1, la survie était de 1,2 an. Cette survie passe à 0,34 an pour les malades ayant un PS 3 ou 4 (p = 0,012). Dans notre étude nous avons retrouvé également une nette dégradation de la médiane de survie avec la sévérité de PS avec une différence statistique significative. La présence d’une anémie lors du diagnostic du cancer bronchique est fréquente, concernant jusqu’à 34% des patients. Son influence sur la survie est variable selon les études. La thrombocytose est assez fréquente dans le CBNPC surtout au stade métastatique [32]. Un taux élevé de plaquette constitue un facteur de mauvais pronostic puisque les plaquettes favorisent l’angiogenèse tumorale [32]. Dans notre étude l’anémie et la thrombocytose étaient associés à un pronostic plus réservé mais sans différence significative. Les marqueurs de l’inflammation ont un effet important sur la survie surtout la CRP [33-35]. La méta-analyse de Jin ayant inclus 8 études a montré qu’une CRP élevée a un impact défavorable sur la survie [36]. En fait, une CRP élevée dans le cadre d’une réponse inflammatoire systémique, en dehors d’un contexte infectieux, reflète un état nutritionnel médiocre, un stade avancé de la maladie et ces patients ont souvent une mauvaise tolérance du traitement et y répondent mal [32,37]. Dans notre série, l’élévation de la CRP était associée à un pronostic plus réservé en étude univariée et multivariée avec un HR de 4,152.

Dans la littérature peu d’études ont analysé la survie des jeunes en fonction du type histologique. L’étude de Skarin [22] a analysé la survie en fonction de différentes formes histologiques du CBP. Dans cette étude le type histologique n’a pas influencé la survie des patients porteurs de CBP, soit un résultat similaire à celui retrouvé dans notre série. Le stade du CBP notamment le CBNPC était le paramètre le plus fortement corrélé à la survie dans différentes études avec une survie significativement meilleure dans les stades précoces (I et II) [7,20,22,23,25,27,38]. Dans l’étude de Bourke *et al.* [27], la survie à 5 ans était 5 fois plus élevé si les malades avaient un stade I ou II par rapport à ceux ayant un stade III ou IV. Nos résultats étaient similaires à ceux de la littérature avec une survie significativement meilleure chez les patients ayant une tumeur localisée par rapport à ceux ayant une tumeur métastatique. Malgré la taille de l’échantillon de notre étude et la disponibilité des données ayant permis d’étudier les facteurs pronostiques du CBNPC chez le sujet jeune, notre travail n’est pas sans limites. En effet, certains facteurs environnementaux ou professionnels pouvant être impliqués dans la genèse du CBP n’ont pas pu être étudier vu la nature de l’étude qui est rétrospective. D’autre part, les déterminants génétiques associés au CBP ainsi que les différentes mutations retrouvées au cours du CBNPC ne sont pas étudiés vu la période d’inclusion des malades (1990 - 2016). Ces mutations ont des implications thérapeutiques et même pronostiques [10-12,25].

## Conclusion

Dans cette étude rétrospective, un indice PS ≥ 2 et une CRP élevée étaient les deux facteurs indépendants associés à un mauvais pronostic du CBNPC chez le sujet jeune. Malgré les progrès thérapeutiques, le CBNPC du sujet jeune demeure de mauvais pronostic. Un diagnostic et une prise en charge précoces permettent d’améliorer la survie de ces patients. En absence de traitement réellement efficace particulièrement aux stades métastatiques, la meilleure stratégie thérapeutique demeure la prévention par la lutte contre le tabagisme surtout chez les adolescents et les adultes jeunes. D’autres études sont nécessaires pour une meilleure appréciation des facteurs pronostiques du CBNPC chez le sujet jeune.

### Etat des connaissances actuelles sur le sujet

- Le cancer bronchique non à petites cellules chez le sujet jeune présente des particularités cliniques et évolutives différentes de la population des patients plus âgés;
- Divers facteurs pronostiques du CBNPC chez le sujet jeune ont été étudiés. Ces facteurs diffèrent d’une population à une autre.

### Contribution de notre étude à la connaissance

- A notre connaissance, ce travail constitue l’un des premiers travaux à s’intéresser aux particularités et facteurs pronostiques du CBNPC chez le sujet jeune dans une population africaine;
- Des déterminants simples à préciser tels que l’indice PS de l’OMS et la CRP peuvent prédire de l’évolution du CBNPC chez le sujet jeune.
